# A taxonomically representative strain collection to explore xenobiotic and secondary metabolism in bacteria

**DOI:** 10.1371/journal.pone.0271125

**Published:** 2022-07-14

**Authors:** Evanthia Kontomina, Vasiliki Garefalaki, Konstantina C. Fylaktakidou, Dorothea Evmorfidou, Athina Eleftheraki, Marina Avramidou, Karen Udoh, Maria Panopoulou, Tamás Felföldi, Károly Márialigeti, Giannoulis Fakis, Sotiria Boukouvala

**Affiliations:** 1 Department of Molecular Biology and Genetics, Democritus University of Thrace, Alexandroupolis, Greece; 2 Department of Chemistry, Aristotle University of Thessaloniki, Thessaloniki, Greece; 3 Department of Medicine, Democritus University of Thrace, Alexandroupolis, Greece; 4 Department of Microbiology, ELTE Eötvös Loránd University, Budapest, Hungary; 5 Institute of Aquatic Ecology, Centre for Ecological Research, Budapest, Hungary; Babasaheb Bhimrao Ambedkar University, INDIA

## Abstract

Bacteria employ secondary metabolism to combat competitors, and xenobiotic metabolism to survive their chemical environment. This project has aimed to introduce a bacterial collection enabling comprehensive comparative investigations of those functions. The collection comprises 120 strains (*Proteobacteria*, *Actinobacteria* and *Firmicutes*), and was compiled on the basis of the broad taxonomic range of isolates and their postulated biosynthetic and/or xenobiotic detoxification capabilities. The utility of the collection was demonstrated in two ways: first, by performing 5144 co-cultures, recording inhibition between isolates and employing bioinformatics to predict biosynthetic gene clusters in sequenced genomes of species; second, by screening for xenobiotic sensitivity of isolates against 2-benzoxazolinone and 2-aminophenol. The co-culture medium of *Bacillus siamensis* D9 and *Lysinibacillus sphaericus* DSM 28^T^ was further analysed for possible antimicrobial compounds, using liquid chromatography-mass spectrometry (LC-MS), and guided by computational predictions and the literature. Finally, LC-MS analysis demonstrated *N-*acetylation of 3,4-dichloroaniline (a toxic pesticide residue of concern) by the actinobacterium *Tsukamurella paurometabola* DSM 20162^T^ which is highly tolerant of the xenobiotic. Microbial collections enable "pipeline" comparative screening of strains: on the one hand, bacterial co-culture is a promising approach for antibiotic discovery; on the other hand, bioremediation is effective in combating pollution, but requires knowledge of microbial xenobiotic metabolism. The presented outcomes are anticipated to pave the way for studies that may identify bacterial strains and/or metabolites of merit in biotechnological applications.

## Introduction

Microorganisms demonstrate diverse metabolic activities that enable them to both tolerate and modify their chemical environments [1]. They employ xenobiotic metabolism to detoxify harmful exogenous chemicals [2] and secondary metabolism to chemically attack their competitors [3]. The two metabolic processes share certain enzymatic components and employ specific acyl-CoA products of acetate and propionate metabolism [4, 5]. Thus, they are believed to have overlapping evolutionary histories, likely linked to fatty acid biosynthesis [4, 6].

The products of microbial secondary metabolism have been recognised for their medicinal utility since ancient times and they remain at the core of pharmaceutical research to date, particularly with relation to antibiotic discovery [3, 7, 8]. Also known as natural products, microbial secondary metabolites (SMs) are bioactive compounds, often with sophisticated chemical structures, that are produced through complex enzymatic pathways which vary widely per specific metabolite and producer organism [9]. In bacteria, each SM is generated via the coordinated action of a unique biosynthetic apparatus that is genetically coded and regulated by an organized array of genes forming a biosynthetic gene cluster (BGC). Genomic data indicate the presence of multiple BGCs per bacterial genome, but only a small fraction of those has been examined to date [10]. Current metagenomic data suggest that 99% of microbial life on earth remains uncultivated, therefore, the potentially exploitable resources of microbial biosynthesis are vast and as yet unexplored [11].

The diversification and adaptability of xenobiotic metabolism, on the other hand, enables microbial survival even under the most adverse of exogenous chemical influences [2, 12]. This remarkable capability renders microorganisms invaluable allies in human efforts to clean polluted environments. Microorganisms are, therefore, employed in bioremediation regimens to eliminate toxic contaminants, particularly the persistent by-products of industry, farming and other human activities [13, 14]. The explosion of genomics has generated immense opportunity to investigate the evolution and function of microbial xenobiotic metabolism.

A major bottleneck to elucidating and exploiting the microbial potential for SM biosynthesis or xenobiotic detoxification is the fact that the corresponding metabolic functions are typically activated only when the appropriate exogenous stimulus is present. Thus, BGCs often remain silent in the absence of microbial competition, while xenobiotic exposure is usually a prerequisite for induction of xenobiotic metabolizing enzymes. Under laboratory conditions, microbial competition can be enforced via the co-culture of isolates [15]. This simple and affordable approach has become popular [16–20], as it allows efficient screening of large collections of microbial isolates, followed by chemical analysis of co-culture media for possible antimicrobial compounds released by the prevailing species. Similarly, microbial collections can be useful for screening of various isolates against specific classes of harmful xenobiotic compounds, monitoring their biotransformation and detoxification via chemical analysis of the culture media.

Taxonomically representative strain collections are considered as key biological resources for studies aiming to elucidate the biosynthetic and/or xenobiotic metabolizing capabilities of microorganisms [21, 22]. However, very large collections, consisting of many thousands of bacterial isolates, are less accessible to the average academic laboratory, as their screening requires automation and infrastructure that are not readily available. Here, we introduce a bacterial collection that includes 120 isolates representing the phyla of *Proteobacteria*, *Actinobacteria* and *Firmicutes*. We demonstrate utility of this collection, as follows: first, by binary co-culture screening of isolates, recording antagonistic interactions and employing bioinformatics to predict possible BGCs; second, by xenobiotic sensitivity screening of isolates to assess biotransformation. Two examples are then presented: first, a specific pair of isolates was subjected to co-culture and the medium was chemically analysed for possible antimicrobial SMs released during competition; second, one specific isolate was challenged with a recalcitrant pesticide residue of concern, and the xenobiotic-amended culture medium was subsequently analysed for possible metabolic detoxification products.

## Materials and methods

### Microbiological material and growth conditions

**Fig 1:**
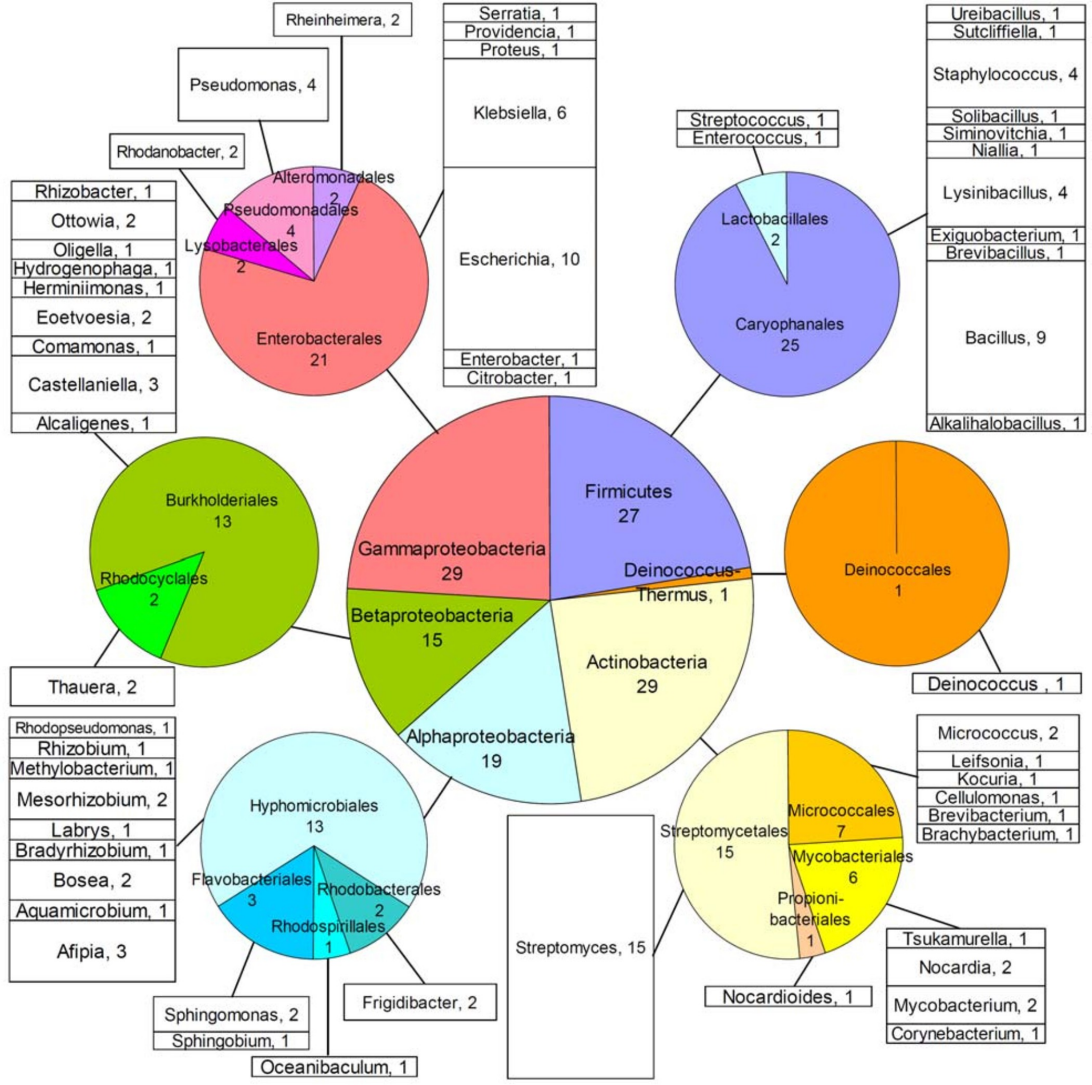
The bacterial collection. Distribution of 120 isolates in higher taxa (central pie), classes (peripheral pies) and genera (stacked columns). Details of isolates are provided in Table A in S1 File.

The microbial collection presented in this study comprised 120 bacterial isolates, assigned serial numbers for laboratory archiving purposes (Fig 1 and Table A in S1 File). Those serial numbers are also used in the present article to facilitate reference to each individual isolate. Seventy-six strains (assigned serial numbers #1–9, #24, #26, #29–31 and #33–94) were microbiologically characterized material available in-house at the Department of Microbiology of Eötvös Loránd University (ELTE, Budapest, Hungary). Those isolates had been collected from various natural or polluted environments, during earlier field studies of the Hungarian group, and their origin is described in Table A in S1 File. An additional 17 strains (#10–23, #25, #27 and #28), had originally been obtained from public repositories, namely the Leibniz Institute German Collection of Microorganisms and Cell Cultures (DSMZ, Braunschweig, Germany) or the American Type Culture Collection (ATCC, Manassas, VA, USA), and these were also mainly of environmental origin. The remaining 27 strains (#101–125, #127 and #128) were microbiologically characterized isolates of clinical origin (Table A in S1 File), available in-house at the Department of Medicine of Democritus University of Thrace (MED-DUTH, Alexandroupolis, Greece).

The original isolates were retrieved either from frozen stocks (isolates #1–28, #57–76 and #101–128), or as single colonies on agar (isolates #29–56), or from lyophilized cultures (isolates #77–94). Isolates were initially incubated for at least 3 d (depending on growth rate) in appropriate 5 mL liquid media (28°C, no shaking), followed by two passages on suitable agar media for selection of pure colonies. Those were finally grown in 5 mL liquid media for preparation of 25% v/v glycerol stocks, archived and permanently frozen at -80°C. The media used for cultivation of each strain are shown in Table A in S1 File, with their composition provided in Table B in S1 File.

### Genomic DNA isolation and 16S rRNA gene amplification and sequencing

All DNA work took place in a sterile cabinet. Genomic DNA was extracted from 5 mL liquid cultures of bacterial isolates, using the DNeasy Blood & Tissue Kit (Qiagen, Hilden, Germany). Protocol adjustments for lysis of Gram-negative or Gram-positive bacteria were according to the manufacturer’s instructions. Almost the entire 16S rRNA gene was amplified [23], with annealing temperature at 48°C. For GC-rich genomes of streptomycetes, suitable primers (Str16S-F: 5’-GCAATCTGCCCTKCACTCTGGGACAAG-3’ and Str16S-R: 5’-CTTCGGGTGTTACCGACTTTCGTGAC-3’) were designed to amplify a major part of the 16S rRNA gene (approx. nucleotide position 120–1420), with annealing temperature at 65°C. Each PCR (50 μl) contained 1.25 U GoTaq thermostable DNA polymerase in 1x Green flexi buffer with 2 mM MgCl_2_ (Promega, Madison, WI, USA), 0.2 mM dNTPs (Takara, Shiga, Japan), 25 pmol each primer (VBC Biotech, Vienna, Austria) and 1 μl (3–250 ng) genomic DNA. The PCR program was initiated at 98°C (5 min), followed by 32 amplification cycles (94°C for 30 s; suitable annealing temperature for 30 s; 72°C for 90 s) and ending at 72°C (10 min). Gel-purified amplification products were Sanger sequenced with PCR primers by Macrogen (Seoul, South Korea) or GATC Biotech (Constance, Germany). Sequences were deposited into the European Nucleotide Archive (ENA) with Project ID: PRJEB47054 (Table A in S1 File).

### Bacterial co-culture screens

Bacterial monocultures in 5 mL of Nutrient Broth (NB) were used to initiate binary co-cultures in 24-well plates with 1 mL per well of Nutrient Agar (NA). The first isolate was inoculated at one side of the well and incubated for 1–3 d, until growth was visible. The second isolate was inoculated diametrically opposite to the first, so that there was no contact between the two isolates (approximately 0.5 cm distance). Monocultures of each isolate were also prepared in separate wells of the plate, to serve as growth controls. Visual inspection of co-cultures was performed daily (over 7 d), to observe possible macroscopic patterns of microbial interaction (e.g. growth inhibition or changes in pigmentation), compared with the respective monocultures.

For a subset of the bacterial collection, co-cultures were performed in 60 x 15 mm Petri dishes. Each co-culture was used to test possible visible antagonistic effects between different pairs of isolates. A representative arrangement of isolates co-cultured in Petri dishes is presented in the Results section. Co-cultures were visually assessed daily (over 10 d).

### Bacterial xenobiotic sensitivity screens

In order to assess their xenobiotic sensitivity, microbial isolates were grown in Petri dishes (100 x 20 mm) containing solid NA media amended either with 2-benzoxazolinone (BOA, PubChem ID 6043; 500 or 1000 μg/mL) or 2-aminophenol (2AP, PubChem ID 5801; 250 μg/mL), both chemicals from Sigma-Aldrich (St. Louis, MO, USA). The final concentration of ethanol in cultures was 1% v/v. In each dish, 5–7 different isolates were tested, with inocula (2 x 10 μl loops from 5 mL starter cultures) placed on the agar medium as parallel lines with approximately 1.5 cm distance between them. The cultures were visually inspected for growth daily, over 10 d. Those screens were repeated as bacterial monocultures grown under the same conditions in 96-well plates, inoculated with one 10 μl loop from each starter culture.

### Extracts of growth medium from bacterial co-culture

The co-culture of choice was grown for 48 h (with shaking) in liquid NB medium, after mixing mid-log phase (OD 600 nm) monocultures of the dominant and inhibited isolates in a 3:1 ratio (final volume 5 mL). After centrifugation at 1500 *g* for 30 min, the supernatant of each culture was filter-sterilized (syringe filters 0.2 μm) and extracted successively (3 times) with 5 mL of hexanes, ethyl acetate and dichloromethane. All solvents were of analytical grade purity. The two liquid monocultures, as well as the medium alone, were also extracted in the same way. The combined 15 mL extracts with each organic solvent were dehydrated with Na_2_SO_4_ (~2.5 g), filtered through Whatman paper and dried by evaporation.

### Extracts of growth medium from xenobiotic-amended bacterial cultures

For assessing xenobiotic metabolism, four liquid cultures (NB, 5 mL) were prepared and grown (with shaking) to mid-log phase (OD 600 nm). Xenobiotic 3,4-dichloroaniline (3,4-DCA, PubChem ID 7257; Sigma-Aldrich, St. Louis, MO, USA) was added at 0, 100, 400 or 800 μg/mL concentration in 5% v/v dimethylsulfoxide (DMSO), and incubation continued for 48 h. A medium-only control (5% v/v DMSO) was also included in the analysis. Following culture centrifugation (1500 *g*, 30 min) and filter-sterilization of the supernatant, the medium’s organic content was extracted successively (3 times) with 5 mL dichloromethane and ethyl acetate. The combined 15 mL extracts with each solvent were dehydrated and dried, as described above.

### Chemical analysis of extracts

For analysis by liquid chromatography-mass spectrometry (LC-MS), a sample of each dried residue was dissolved in methanol (LC-MS grade) and filtered through a PTFE syringe filter (pore size 0.45 μm, diameter 13 mm). LC-MS analysis took place on a Shimadzu LC-MS 2010 EV system under electrospray ionization (ESI) conditions, using a reverse phase column with methanol as the eluent. Collected fragments were examined within a range from 50 to 600 Da, and the elution time was 0 to 20 min.

### Computational methods

For the molecular genetic classification of bacterial isolates, sequencing of 16S rRNA genes was followed by interrogation of the EzBioCloud database [24] to determine the closest related species/strain. Species names are according to the List of Prokaryotic Names with Standing in Nomenclature (https://lpsn.dsmz.de/). Nucleotide identification numbers (Table A in S1 File) were obtained from the ENA (Project ID: PRJEB47054) and Taxonomy identifiers (TAXIDs) were according to the Taxonomy database (https://www.ncbi.nlm.nih.gov/taxonomy).

Bacterial genome mining for BGCs was conducted using antibiotics and Secondary Metabolite Analysis Shell (antiSMASH) software version 5.0 [25], enabling antiSMASH 6 beta features, ClusterBlast and MIBiG cluster comparison. The reference genome for each taxonomically classified strain (or close relative) of the bacterial collection was surveyed, and the GenBank files of all putative BGCs found were downloaded and saved as individual files locally. All *in silico* analyses were performed during academic year 2020–2021.

## Results

### The bacterial collection

The collection comprises 120 bacterial isolates (Fig 1 and Table A in S1 File), classified microbiologically and by 16S rRNA gene sequencing to represent different taxonomic groups. Specifically, the collection includes 56 Gram-positive isolates (29 *Αctinobacteria* and 27 *Firmicutes*), as well as 63 Gram-negative *Proteobacteria* (19 *Alphaproteobacteria*, 15 *Betaproteobacteria* and 29 *Gammaproteobacteria*), and one *Deinococcus* (*D*. *ficus*). Combined, the isolates belonged to 17 classes, 59 genera and 92 species. The majority (isolates #1–94) are non-pathogenic free-living bacteria of environmental origin, while a subset (isolates #101–128) originated from human clinical samples. Those isolates were, therefore, chosen on the basis of their broad taxonomic range, laboratory utility and environmental, biotechnological, and/or clinical relevance, as well as their postulated biosynthetic and/or xenobiotic detoxification capabilities. Fifteen species were represented by more than one isolate, for the purposes of validating the outcomes of experimental procedures and to enable future studies of polymorphisms in specific genes of interest. Most isolates belonged to, or were related to, species with sequenced genomes in the GenBank database. On the basis of those criteria, the collection was considered as suitable for laboratory investigations of biological processes relevant to secondary and/or xenobiotic metabolism, and two lines of investigation were initiated as described below.

### Screening for antagonistic interactions between co-cultured bacterial isolates

#### Binary co-culture screen of the collection

To demonstrate the utility of the compiled collection for studies of antagonistic interactions between bacteria, we have undertaken 4992 binary co-cultures of isolates in different combinations, spanning the full taxonomic spectrum available (Table 1 and Table C in S1 File). Distance microbial assays were performed on solid medium, using routine culture conditions and a 24-well plate format that enabled efficient screening and easy visualization of possible phenotypic changes caused by microbial competition. Antagonistic interactions were observed for 289 combinations of co-cultured bacteria (Table 1 and Table C in S1 File), where growth of the second inoculated isolate was impaired. No apparent macroscopic changes were observed in colony morphology or pigmentation.

**Table 1:** Summary of the bacterial co-culture screen and observed growth inhibition^a^.

	Isolate inoculated first												Total	
	*Firmicutes*		*Deinococcus-Thermus*		*Actinobacteria*		*Alphaproteobacteria*		*Betaproteobacteria*		*Gammaproteobacteria*			
**Isolate inoculated second**	Co-cultures	Inhibition	Co-cultures	Inhibition	Co-cultures	Inhibition	Co-cultures	Inhibition	Co-cultures	Inhibition	Co-cultures	Inhibition	Co-cultures	Inhibition
*Firmicutes*	306	51	18	0	236	30	84	8	112	13	431	10	**1187**	**112**
*Deinococcus-Thermus*	18	2	0	0	14	1	0	0	7	0	0	0	**39**	**3**
*Actinobacteria*	212	46	14	1	166	9	165	4	56	0	357	9	**970**	**69**
*Alphaproteobacteria*	90	18	9	0	165	12	272	7	92	1	405	5	**1033**	**43**
*Betaproteobacteria*	91	25	7	1	84	11	105	10	36	0	24	1	**347**	**48**
*Gammaproteobacteria*	401	8	25	0	108	0	156	0	24	0	702	6	**1416**	**14**
**Total**	**1118**	**150**	**73**	**2**	**773**	**63**	**782**	**29**	**327**	**14**	**1919**	**31**	**4992**	**289**

^a^The complete dataset is provided in Table C in S1 File.

#### Binary co-culture screen of *Tsukamurella paurometabola* vs. other bacteria

**Fig 2:**
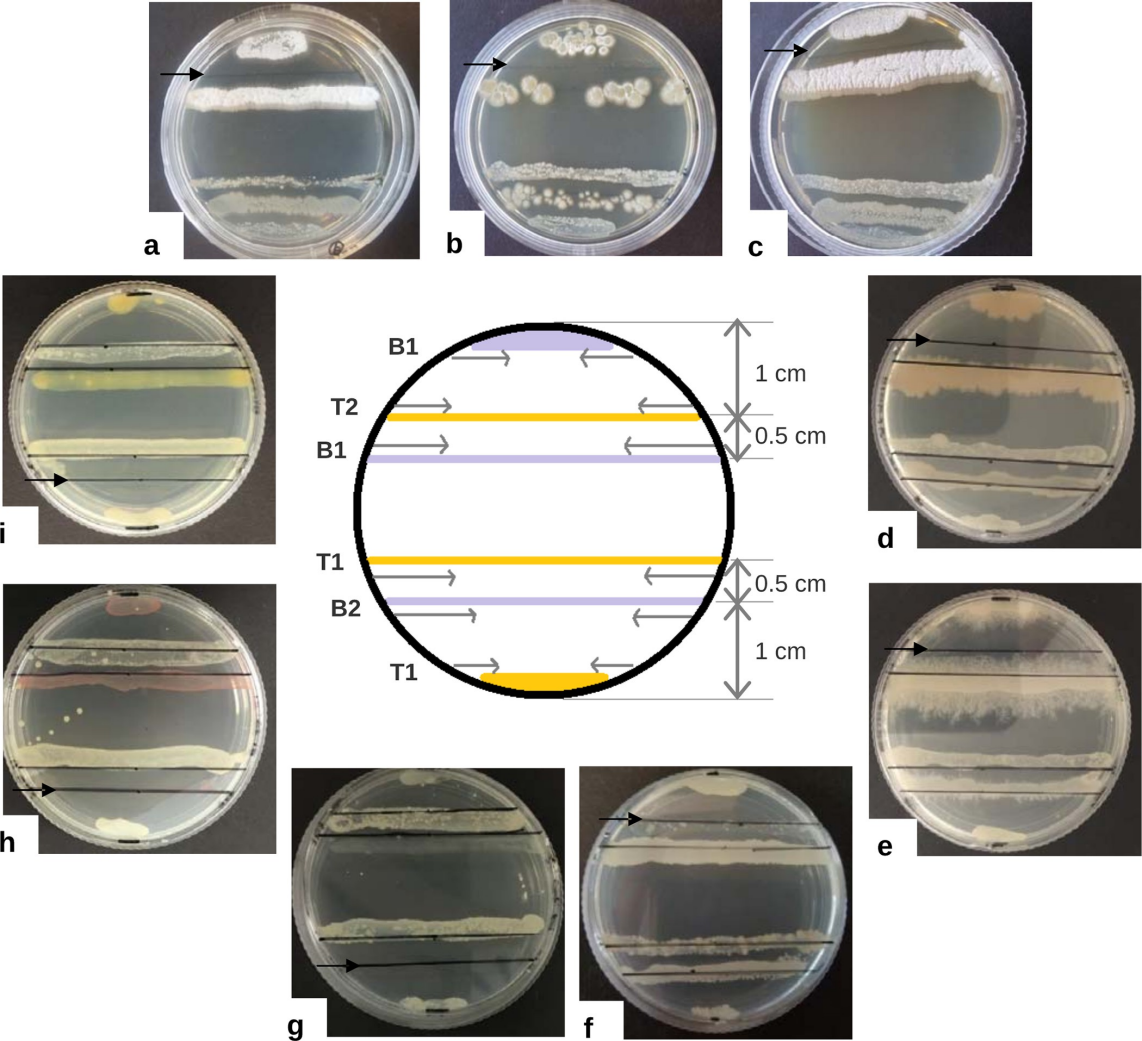
Examples of competition between *Tsukamurella paurometabola* and other bacterial isolates of the collection. Co-culture of isolate #25-*T*. *paurometabola* DSM 20162^T^ was performed according to the arrangement illustrated in the centre, as follows: each bacterial isolate was initially inoculated at diametrically opposite parts of a 60 x 15 mm Nutrient Agar (NA) plate. *T*. *paurometabola* was inoculated along the lines labelled T1, while the second bacterium was inoculated along the lines labelled B1. The two isolates were incubated until growth was readily visible. Subsequently, a second round of inoculation took place, with *T*. *paurometabola* inoculated along the line labelled T2 and the other bacterium inoculated along the line labelled B2. The approximate distance between those inoculated lines is indicated. Visual inspection of co-cultures took place daily, over a period of 10 d. Inoculation was performed using a sterile loop twice, and the isolates were spread along the lines in the direction indicated by the horizontal arrows. Representative results of this procedure are presented, as follows: clockwise, binary co-cultures with isolates #84-*Streptomyces griseus* subsp. *griseus* FBUA 801 (a), #87-*Streptomyces* sp. FBUA 1287 (b), #89-*Streptomyces zaomyceticus* FBUA 1571 (c), #26-*Lysinibacillus sphaericus* ZK38 (d), #30-*Bacillus licheniformis* B3 (e), #35-*Bacillus siamensis* D9 (f), #16- *Sphingomonas sanxanigenens* DSM 19645^T^ (g), #53-*Hydrogenophaga carboriunda* TV-122 (h) and #74-*Rhodanobacter lindaniclasticus* RB3-4A (i). The photographs were taken 7 d after incubation of the co-cultures and the black arrows indicate the inoculation line of the inhibited isolate. *T*. *paurometabola* is inhibited by the *Actinobacteria* (a-c) and the *Firmicutes* (d-f), but inhibits the *Proteobacteria* (g-i).

The actinobacterium *T*. *paurometabola* DSM 20162^T^ (isolate #25) was specifically subjected to binary co-culture against a subset of 76 taxonomically representative bacteria of the collection, according to the scheme described in Fig 2, paving the way toward more comprehensive investigation of the inhibition patterns observed during the competition of isolates. In each Petri dish, the arrangement of co-cultures allowed simultaneous assessment of *T*. *paurometabola* DSM 20162^T^ influence on growth of the competitor strain and vice versa, with examples shown in Fig 2.

Antagonistic interactions were observed in 52 of the 152 combinations tested, with complete inhibition exerted by the corresponding dominant isolate in 22 cases (Table 2 and Table D in S1 File). *T*. *paurometabola* DSM 20162^T^ appeared to confer an inhibitory effect mainly on proteobacteria, particularly alphaproteobacteria where growth of six isolates (#2, #3, #7, #16, #18 and #59) was totally impaired. Although the bacterium was an effective competitor of several actinobacteria, most notably of mycobacteria (isolates #1 and #8), it was however negatively impacted by streptomycetes. Specifically, *T*. *paurometabola* DSM 20162^T^ was completely inhibited by five (isolates #78, #84, #87, #89 and #90) of the nine streptomycete isolates tested. Several *Bacilli* also prevented growth of *T*. *paurometabola* DSM 20162^T^, with total inhibition conferred by *Lysinibacillus sphaericus* (isolates #22, #24 and #26) and *Bacillus cereus* (isolate #33) (Fig 2, Table 2, and Table D in S1 File).

**Table 2:** Summary of the bacterial co-culture screen for the actinobacterium *Tsukamurella paurometabola*^a^^,^^b^.

	*Firmicutes*		*Deinococcus-Thermus*		*Actinobacteria*		*Alphaproteobacteria*		*Betaproteobacteria*		*Gammaproteobacteria*		Total	
	Co-cultures	Inhibition	Co-cultures	Inhibition	Co-cultures	Inhibition	Co-cultures	Inhibition	Co-cultures	Inhibition	Co-cultures	Inhibition	Co-cultures	Inhibition
*T*. *paurometabola* DSM 20162^T^ inoculated first	20	3(1)	1	0	21	7(4)	15	9(6)	12	5(1)	7	5(1)	**76**	**29(13)**
*T*. *paurometabola* DSM 20162^T^ inoculated second	20	9(4)	1	0	21	9(5)	15	3(0)	12	2(0)	7	0	**76**	**23(9)**

^a^Co-cultures demonstrating substantial inhibition (in parentheses, instances of total inhibition).

^b^The complete dataset is provided in Table D in S1 File.

#### Bacterial genome mining for BGCs

**Fig 3:**
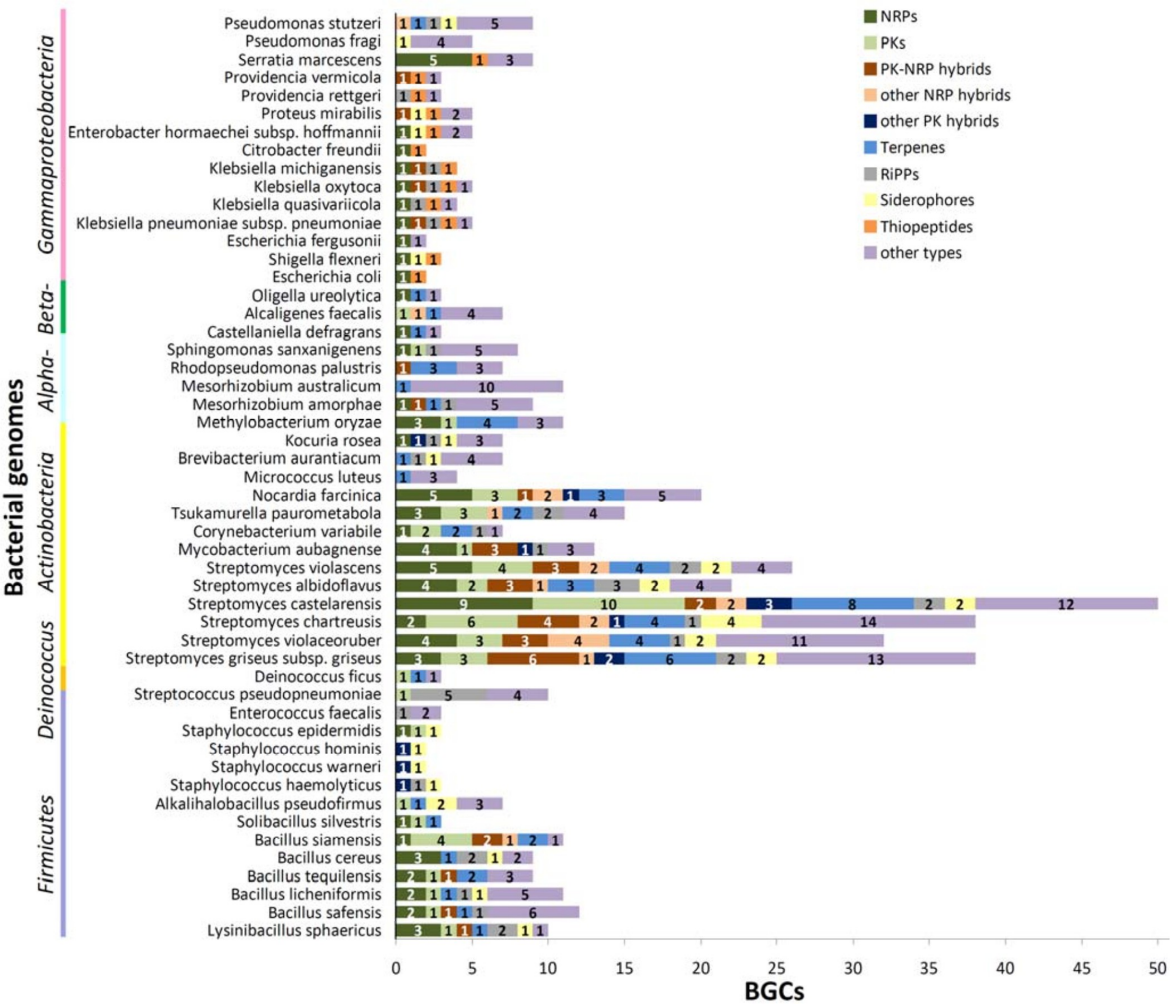
Distribution of biosynthetic gene clusters (BGCs) predicted in complete sequenced genomes of bacterial species represented in the collection. The graph illustrates the distribution of clusters for non-ribosomal peptides (NRPs), polyketides (PKs), PK-NRP hybrids, other NRP or PK hybrids, terpenes, ribosomally synthesized and post-translationally modified peptides (RiPPs), siderophores, thiopeptides, or "other types".

To further explore the utility of our bacterial collection in studies of secondary metabolism, we looked for species with completely sequenced genomes (deposited in the GenBank database) and subjected them to computational analyses aiming to identify putative BGCs. Genomic analysis of 51 sequenced bacterial species matching our collection identified 502 putative BGCs in total (Fig 3 and Table E in S1 File). Of those, 279 BGCs were predicted in the 13 analysed species of *Actinobacteria*, mainly streptomycetes (206 BGCs) which were found to possess the highest number (22 to 50) of BGCs per genome. In contrast, species belonging to the orders *Burkholderiales* (class *Betaproteobacteria*), *Enterobacterales* (class *Gammaproteobacteria*) and *Deinococcales* (class *Deinococci*) were found to carry the smallest number (2 to 9) of BGCs per genome (Fig 3 and Table E in S1 File). Although putative BGCs localized almost exclusively to the bacterial chromosomes, three of them were found in plasmids isolated from *Mesorhizobium amorphae* CCNWGS0123, *T*. *paurometabola* DSM 20162^T^, and *Alkalihalobacillus pseudofirmus* OF4 (Table E in S1 File).

The predicted BGCs corresponded to 10 major types, classified as clusters for non-ribosomal peptides (NRPs), polyketides (PKs), PK-NRP hybrids, other NRP or PK hybrids, terpenes, ribosomally synthesized and post-translationally modified peptides (RiPPs), siderophores, thiopeptides, or "other types". Among them, clusters for NRPs (77 BGCs), terpenes (62 BGCs) and PKs (53 BGCs) were the most common. The type and distribution of predicted BGCs varied significantly between bacterial genomes belonging to different taxonomic groups. For example, BGCs for thiopeptides were identified only in *Gammaproteobacteria*, while BGCs for siderophores were found in all taxonomic groups except *Alphaproteobacteria* and *Betaproteobacteria* (Fig 3 and Table E in S1 File).

The results obtained were also compared to the MIBiG database entries providing information about experimentally investigated microbial BGCs reported in the literature. Of the BGCs predicted, approximately 22% (112 BGCs) demonstrated at least 70% genomic similarity to previously characterized clusters, facilitating inference of at least the core structure of putative SMs. The detailed results of MIBiG analyses are provided in S2 File.

#### LC-MS analysis of co-cultured pair of isolates

The co-culture screen of the bacterial collection identified isolate #35-*Bacillus siamensis* D9 as particularly "aggressive", inhibiting growth of 29 other isolates tested, including 11 of 17 co-cultured *Firmicutes*, 7 of 15 *Actinobacteria* (but not streptomycetes), 6 of 9 *Alphaproteobacteria*, 3 of 7 *Betaproteobacteria*, 1 of 24 *Gammaproteobacteria* and the single *Deinococcus* (Tables C and D in S1 File). To investigate whether co-culture might induce the production of SMs with antimicrobial potential, isolates #35-*B*. *siamensis* D9 and #22-*L*. *sphaericus* DSM 28^T^ were grown together, followed by organic extraction of the medium and LC-MS analysis. The respective monocultures, as well as a medium-only sample were used for comparison. All LC-MS peaks (positive and negative ionization) were recorded for extracts with all three solvents employed. Peaks specific to the culture medium were then subtracted from the results of the two monocultures and the co-culture. The remaining peaks were compared between the co-culture and its respective monocultures, allowing identification of molecules likely generated through the antagonistic interaction of the two isolates.

A total of 27 fragments with masses below 600 Da (56 to 564.1 Da) were recorded as unique to the co-culture medium (Table F in S1 File). Additional fragments were recorded as potentially matching bibliographically described SMs [26–29], produced by species of the *Bacillus amyloliquefaciens* **group which includes** *B*. *siamensis* **[30].** Two of the fragments found exclusively in the co-culture medium extract had masses of 565.2 and 587.2 Da (retention time [r.t.] 7–8 min), probably corresponding to macrolactin Q [M+H]^+^ and [M+Na]^+^, respectively. An ion of 402.9 Da (r.t. 4–5 min) likely corresponded to C_17_-bacillomycin-D or macrolactin H. Another ion with a mass of 540.9 Da (r.t. ~10 min) matched fragments characteristic of fengycins A (C_15_, C_16_, C_17_), while a daughter fragment of surfactin was predicted at 483 Da (r.t. 12–13 min) **(Table 3)**. Finally, two fragments at 196.9 Da (r.t. 4–5 min) and 327.7 Da (r.t. around 12 min) were found both in the monoculture of *B*. *siamensis* and its co-culture with *L*. *sphaericus*, and those probably corresponded to phenaminomethylacetic acid [M+CH_3_OH]^‒^ [31] and its enzymatically produced glucoside **(Table 3)**.

**Table 3:** Detected LC-MS peaks matching chemically characterized compounds, described in the literature as secondary metabolites produced by species of the *Bacillus amyloliquefaciens* group that includes *Bacillus siamensis*^a^.

Compound	Exact mass (Da)	Medium	Monoculture of isolate #35^b^	Monoculture of isolate #22	Co-culture of isolates #35 with #22^b^
Phenaminomethylacetic acid [M+CH_3_OH]^‒^	197.1	Not detected	Detected (intensity x10,000)	Not detected	Detected (intensity x10,000)
			m/z: 196.9; r.t.: 5–4; Hexanes (E-)		m/z: 196.9; r.t.: 5–4.5; Dichloromethane (E-)
Glucoside of phenaminomethylacetic acid	328.1	Not detected	Detected (intensity x1,000)	Not detected	Detected (intensity x10,000)
			m/z: 327.7; r.t.: 14.5–13.9; Hexanes (E-)		m/z: 327.7; r.t.: 10.2–9.9; Hexanes (E-)
Macrolactin Q [M+H]^+^	565.1	Not detected	Not detected	Not detected	Detected (intensity x1,000,000)
					m/z: 565.2; r.t.: 8–7.4; Dichloromethane (E+)
Macrolactin Q [M+Na]^+^	587.3	Not detected	Not detected	Not detected	Detected (intensity x1,000,000)
					m/z: 587.2; r.t.: 8–7; Ethyl acetate (E+)
Macrolactin Q [M+Na]^+^	587.3	Not detected	Not detected	Not detected	Detected (intensity x1,000,000)
					m/z: 587.2; r.t.: 8–7.4; Dichloromethane (E+)
Macrolactin H or C_17_-Bacillomycin-D	402.2 or 402.3	Not detected	Not detected	Not detected	Detected (intensity x10,000)
					m/z: 402.9; r.t.: 5–4; Ethyl acetate (E-)
Fengycins A (C_15_, C_16_, C_17_)	540.8	Not detected	Not detected	Not detected	Detected (intensity x1,000)
					m/z: 540.9; r.t.: 10.3–9.9; Ethyl acetate (E-)
Surfactin	483.3	Not detected	Not detected	Not detected	Detected (intensity x10,000)
					m/z: 483.0; r.t.: 12.7–12; Ethyl acetate (E+)

^a^The table describes LC-MS peaks detected in the co-culture medium of isolates #35-*Bacillus siamensis* D9 and #22-*Lysinibacillus sphaericus* DSM 28^T^, comparing with the respective monocultures and the culture medium alone. Relevant references are: [26–29,31].

^b^m/z is mass to charge ratio in Da units; r.t. is retention time in min; E+/E- is positive/negative ionization.

### Screening for xenobiotic sensitivity of bacterial isolates

#### Xenobiotic sensitivity screen of the collection with BOA and 2AP

To explore the xenobiotic metabolizing potential of the bacterial isolates in our collection, we undertook sensitivity assays against two compounds of interest: BOA is a natural product, synthesized by grasses as a protective phytoanticipin against pathogens [32]; 2AP has been reported as the intermediate metabolic derivative of BOA detoxification in grass-endophytic fungi that are resistant to the phytoanticipin [33]. Moreover, both compounds are subject to large-scale production as synthetic chemicals with applications in the pharmaceutical and the dye industry [34, 35].

**Fig 4:**
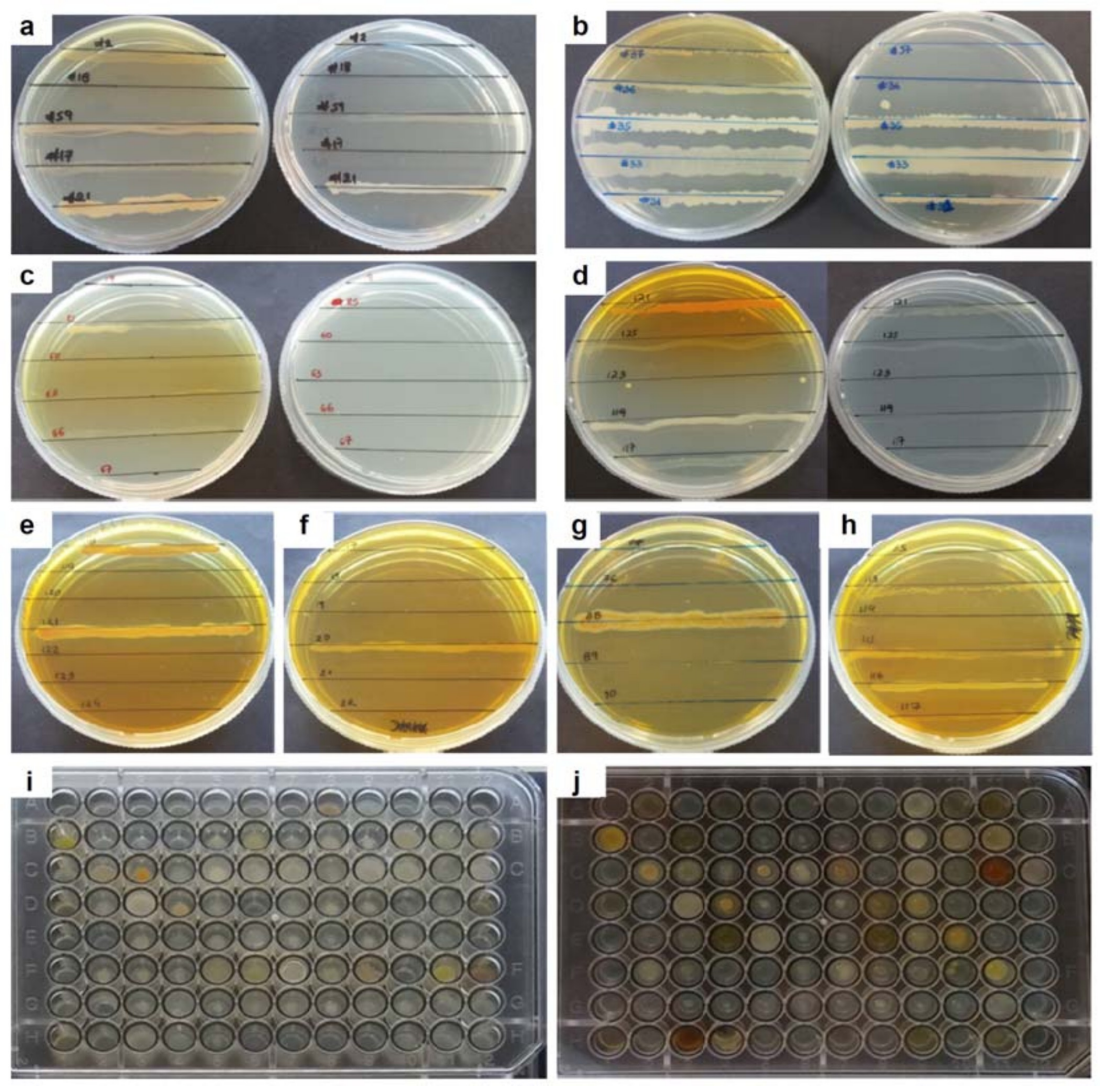
Sensitivity of bacterial isolates to xenobiotics. In a-d, the examples shown are xenobiotic sensitivity screens with 2-benzoxazolinone (BOA) in concentrations of 500 or 1000 μg/mL (Petri dish on the left- or the right-hand side of each image, respectively). In e-h, the examples shown are xenobiotic sensitivity screens with 250 μg/mL of 2-aminophenol (2AP). In i and j, the xenobiotic sensitivity screens were repeated for monocultures grown in 96-well agar plates, and the example shows the results for 96 (of 107 total) bacterial isolates screened in the absence (i) or the presence (j) of BOA (500 μg/mL). Identical screens were also performed with 1000 μg/mL of BOA or 250 μg/mL of 2AP (not shown). In a-d, the displayed bacterial isolates are as follows: in a, isolates #2, #18, #59, #17 and #21; in b, isolates #37, #36, #35, #33 and #31; in c, isolates #19, #25, #60, #63, #66 and #67; in d, isolates #121, #125, #123, #119 and #117; in e, isolates #118, #119, #120, #121, #122, #123 and #124; in f, isolates #17, #18, #19, #20, #21 and #22; in g, isolates #87, #76, #88, #89 and #90; in h, isolates #105, #113, #114, #115, #116 and #117 (Table G in S1 File for details).

A representative set of 107 bacterial isolates was tested for the ability to grow on NA medium containing BOA or 2AP (Fig 4 and Table G in S1 File). The majority (89 isolates), were able to grow on solid media supplemented with 500 μg/mL of BOA. Of those, 68 isolates were also able to survive in cultures containing 1000 μg/mL of the compound. Among bacteria surviving BOA, 14 isolates (12 species of *Proteobacteria*, 1 species of *Actinobacteria* and 1 species of *Firmicutes*) were additionally recorded to apparently metabolize the compound to 2AP, as indicated by the observed change in the coloration (yellow to orange) of the agar medium (Fig 4 and Table G in S1 File). Only 47 bacterial isolates were able to survive in 250 μg/mL of 2AP, and most of them demonstrated very poor growth. The majority of bacteria found to tolerate this concentration of 2AP were *Proteobacteria* (38 isolates), mainly enterobacteria (*Gammaproteobacteria*) where coloration of the cultures became especially intense (Fig 4 and Table G in S1 File).

#### Transformation of 3,4-DCA xenobiotic by *T*. *paurometabola*

**Fig 5:**
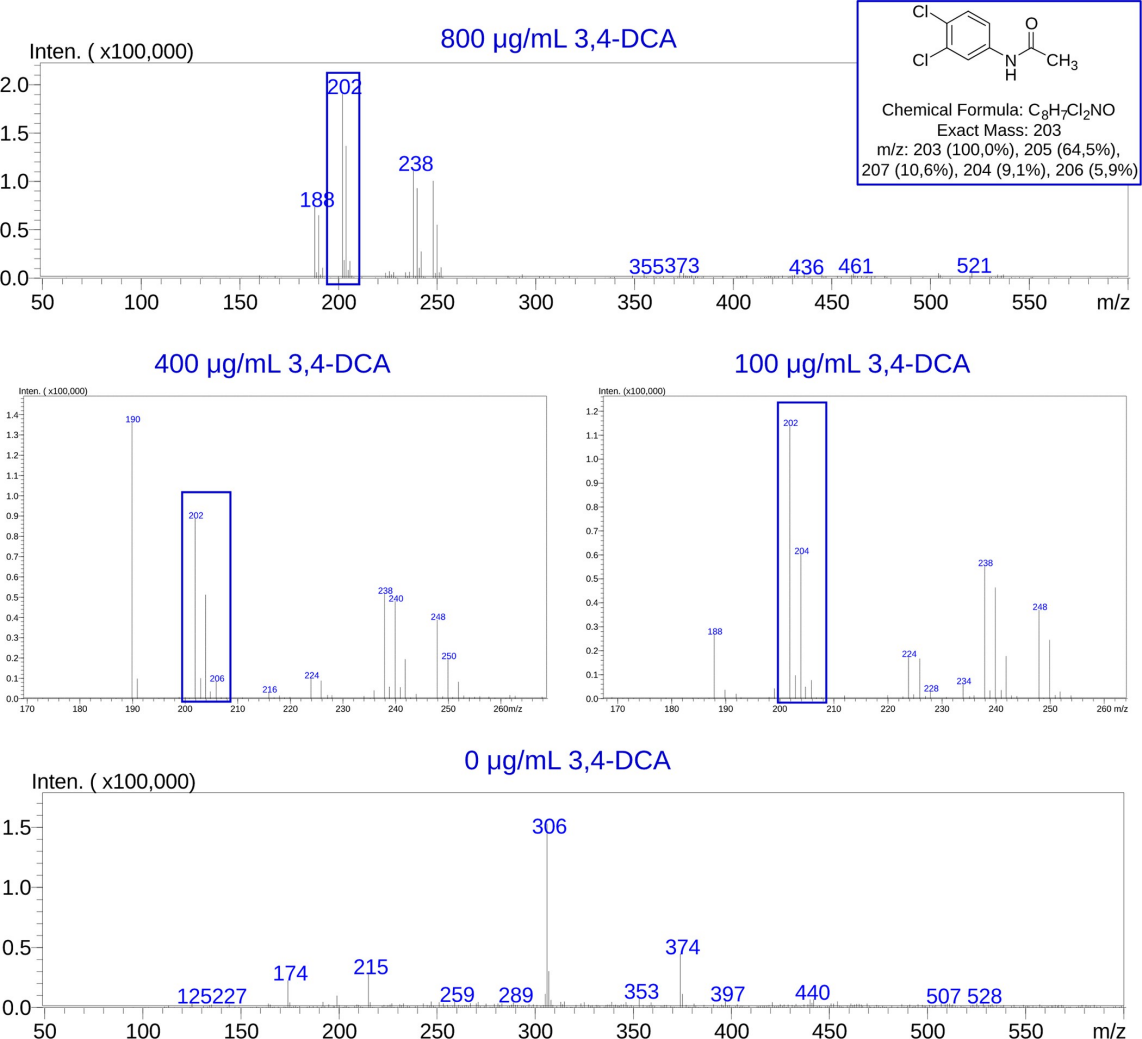
LC-MS analysis of xenobiotic-amended culture medium, following growth of isolate #25-*T*. *paurometabola* DSM 20162^T^. The actinobacterium was allowed to grow in Nutrient Broth (NB) medium amended with 800, 400, 100 or 0 μg/mL of 3,4-dichloroaniline (3,4-DCA). The culture medium was then subjected to organic extraction and LC-MS analysis, to specifically look for possible *N-*acylated metabolic derivatives of the parent compound. Negative ion mode ESI spectrometry detected the characteristic isotopic pattern (peak intensity ratio 9:6:1, retention time ~8.5 min) of a bis-chlorinated compound with mass corresponding to the *N-*acetylated form of 3,4-DCA [M-H]^‒^ (peaks and chemical structure in blue frames). This compound was absent in the extract of the culture that lacked the parent arylamine (0 μg/mL).

*T*. *paurometabola* DSM 20162^T^ (isolate #25) was further chosen to investigate possible biotransformation of 3,4-DCA, a persistent toxic pollutant released as the by-product of propanil and other phenylamide agrochemicals in farmlands [36]. We have become interested in *T*. *paurometabola* DSM 20162^T^ during our earlier studies of microbial 3,4-DCA detoxification through enzymatic conjugation reactions catalyzed by arylamine *N-*acetyltransferases (NATs) [37, 38], as this actinobacterium was found to possess a NAT homolog with unusual properties [37]. Here, *T*. *paurometabola* DSM 20162^T^ was challenged with a concentration range of 3,4-DCA added to the culture medium, which was then subjected to extraction and LC-MS analysis of its organic content for possible *N-*acetylated, *N-*propionylated and/or *N-*malonylated metabolic derivatives. The corresponding authentic compounds had been synthesized previously [37] and were available in-house as standards. Analysis of the medium-only control was used to facilitate the identification of fragments unique to the xenobiotic-amended cultures, particularly those fragments specific to the postulated *N-*acylated derivatives of 3,4-DCA. In the negative ion mode, the characteristic isotopic pattern of a bis-chlorinated compound was detected, demonstrating three distinctive peaks in the molecular ion region of 3,4-dichloroacetanilide [M-H]^‒^ (exact mass 203). Those peaks had 2 m/z units distance between them (M, M+2 and M+4) and their intensity ratio was 9:6:1 (Fig 5). The LC-MS analysis detected only the *N-*acetylated form of 3,4-DCA, and the quantities were roughly proportionate to the concentration range (100, 400, 800 μg/mL) of the parent compound added to the culture medium of the bacterium. The medium of the control culture (0 μg/mL 3,4-DCA) did not contain any bis-chlorinated anlinine, as expected (Fig 5).

## Discussion

In their natural habitat, bacteria release SMs to eliminate or inhibit competitors. At the same time, they employ xenobiotic metabolism to annihilate the chemical threats of their environment via enzymatic detoxification of exogenous substances. Systematic laboratory investigations and comparisons of such metabolic processes across a broad taxonomic spectrum of bacteria can be very difficult, as microbial cells demonstrate huge diversity in their growth requirements, biochemistry and physiology, particularly in regard with their responses to exogenous stimuli. Therefore, we have aimed to compile a practicable bacterial collection, to enable comprehensive "pipeline" screens and comparative investigations relevant to microbial functions of interest. The presented set comprises 120 strains spanning the main taxonomic groups of bacteria. Those strains were retrieved from different environments (mainly aquatic), including natural or polluted sources, as well as from various clinical specimens. The non-pathogenic free-living strains are likely to demonstrate enhanced xenobiotic detoxification capabilities, including towards specific environmental pollutants of interest (e.g. arylamines and other industrial aromatic compounds), thus facilitating environmental investigations relevant to agriculture, biotechnology and bioremediation. Many of those strains (e.g. bacilli and actinobacteria) are also considered as potent producers of SMs, including antimicrobials that could target the pathogenic isolates of the collection, which are often associated with nosocomial infections. Importantly, the number and subsistence requirements of strains allow efficient management of the collection in the common laboratory, without need for sophisticated microbiology equipment or expertise.

To demonstrate the utility of the compiled bacterial collection, two lines of investigation were launched. The first involves co-culture of strains in pairs, looking for antagonistic interactions between them. Given the lack of contact between the co-cultured isolates on agar, the observed inhibitory effects on vulnerable strains are expected to be the outcome of diffusible compounds released in the media by the dominant strains. *Firmicutes* and *Actinobacteria* inhibited growth in 14 and 9% of the tested co-cultures, respectively. Among *Firmicutes*, the most "aggressive" isolates were #35 (*B*. *siamensis*), #22, #24 and #26 (*L*. *sphaericus*), #29 and #38 (*Bacillus* sp.), #31 (*Bacillus tequilensis*) and #33 (*B*. *cereus*). Among *Actinobacteria*, some streptomycetes, particularly isolate #87, caused growth inhibition of several of the isolates tested, followed by isolate #27 (*Brevibacterium aurantiacum*), #47 (*Kocuria rosea*) and others. *Proteobacteria* were less antagonistic, with growth inhibition exerted by *Betaproteobacteria*, *Alphaproteobacteria* and *Gammaproteobacteria* in 4.7, 4 and 1.6% of the tested co-cultures, respectively. It is worth noting that 16 of the 31 inhibitions attributed to *Gammaproteobacteria* were conferred by the enterobacterium *Serratia marcescens* (isolate #121), with its antagonistic effect evident across all tested taxonomic groups, apart from its own (i.e. the *Gammaproteobacteria*). Overall, the *Betaproteobacteria* appeared more sensitive, with growth inhibition observed in 14.8% of the co-culture combinations tested, while *Gammaproteobacteria* appeared the least vulnerable of all isolates.

It is interesting that, while considerable competition was observed among bacteria from various environmental sources, there was little antagonistic interaction between those and the clinical Gram-negative enterobacteria (*Gammaproteobacteria*) of the collection. Although not surprising, given the non-overlapping habitats of those microbial groups, this observation also highlights one of the difficulties in discovering new natural products with antimicrobial activity against Gram-negative pathogens of clinical concern [39]. As corroborated by our *in silico* genome mining for BGCs, the Gram-positive *Actinobacteria* and *Firmicutes* appear to dedicate a good part of their genome and cellular resources to the biosynthesis of SMs; however, evolution has likely favored the production of compounds targeting other free-living competitors, rather than microbes associated with animal hosts [40]. Implementation of additional co-culture schemes would be useful, e.g. by growing the paired isolates within adjoining compartments separated by a dialysis membrane to allow diffusion of small molecules while preventing cell contact, or by performing contact co-cultures, either on agar or by mixing together liquid monocultures [41].

For example, contact co-culture is expected to complement the results obtained from the distance co-cultures performed between *T*. *paurometabola* DSM 20162^T^ and other isolates of the collection. *T*. *paurometabola* DSM 20162^T^ was impacted by the presence of Gram-positive bacilli and streptomycetes, but appeared to prevail over its more closely related (class *Mycobacteriales*) isolates #1-*Mycobacterium aubagnense* FII-6, #8-*Mycobacterium chlorophenolicum* PIII-13 and #92-*Nocardia* sp. FBUA 1711. Like *T*. *paurometabola* DSM 20162^T^, those are all corynebacteria characterized by the presence of mycolic acids in their cell wall [42], and they are encountered in the clinic as opportunistic pathogens of concern [43–46]. Our computational analysis of *T*. *paurometabola* DSM 20162^T^ genome predicts 14 BGCs that could potentially direct biosynthesis of SMs upon competition with other corynebacteria. Furthermore, investigations have demonstrated mycolata bacteria, including *Tsukamurella*, to stimulate the activation of BGCs in streptomycetes, upon contact co-culture in the same liquid medium [47]. These are worthwhile experiments to perform for *T*. *paurometabola* DSM 20162^T^ and other corynebacteria in the collection. Moreover, the outcomes of our ongoing screens could be expanded through the application of modern co-culture technologies [48], such as the iChip device [49].

To pave the way towards future characterization of candidate antimicrobial SMs released by competing isolates of the collection, we compared the organic content of cell-free extracts prepared from the co-culture and respective monoculture media of *B*. *siamensis* D9 and *L*. *sphaericus* DSM 28^T^. Agar competition assays demonstrated both isolates to be potent in antagonizing several bacterial strains, as well as each other. BGC analysis of the available sequenced genomes of *L*. *sphaericus* DSM 28^T^ and *B*. *siamensis* SCSIO 05746 predicted 10 and 11 BGCs, respectively. There was limited bibliographic information about secondary metabolites of *L*. *sphaericus* DSM 28^T^, to allow interpretation of predicted BGCs. In contrast, five of the computationally predicted BGCs in *B*. *siamensis* SCSIO 05746 fully (100%) matched the previously characterized BGCs for bacilysin, difficidin, fengycin, bacillaene and macrolactin H of *Bacillus velezensis* [50], a close relative belonging to the *B*. *amyloliquefaciens* group [30]. There was also 82% match with the BGC for surfactin of *B*. *velezensis* [50], as well as 100% match with the BGC for bacillibactin [51] of the closely related species *Bacillus subtilis* [30]. Although genomic BGC content may vary even among strains of the same species, advanced computational search combined with rigorous survey of the literature can be very informative until the genomic sequences of all bacterial strains of the collection become available.

To look for possible antibacterial compounds, likely to be the products of direct competition between *B*. *siamensis* D9 and *L*. *sphaericus* DSM 28^T^, we assessed the LC-MS fragments detected exclusively in the extract of the co-culture medium. Guided by the aforementioned BGC computational predictions and the literature [26–29], we were able to infer match of some of the detected LC-MS fragments to the polyketides macrolactin Q and H, as well as to the cyclic lipopeptides fengycin A, bacillomycin-D and surfactin. Macrolactins and cyclic lipopeptides are described as bioactive SMs targeting bacteria and fungi, respectively [27, 50]. Therefore, we speculate that the compound conferring the inhibitory effect of *B*. *siamensis* D9 (isolate #35) to other bacteria of the collection is probably macrolactin. Moreover, it is possible that other LC-MS fragments detected in the co-culture medium may represent as yet undescribed SMs released by either of the two competing strains.

The literature also describes phenaminomethylacetic acid as an effective antimicrobial compound, produced by *B*. *velezensis* [31]. To find out whether phenaminomethylacetic acid (PubChem CID: 817923; [31]) was present in the co-culture extract of *B*. *siamensis* D9 and *L*. *sphaericus* DSM 28^T^, the LC-MS results were initially examined for molecular ions that could match the compound. The findings were consistent with possible presence of phenaminomethylacetic acid and its glucoside in the medium extract prepared from both the monoculture of *B*. *siamensis* D9 and its co-culture with *L*. *sphaericus* DSM 28^T^, suggesting constitutive rather than inducible biosynthesis of the metabolite by the former isolate. Phenaminomethylacetic acid is reported as an antifungal agent effective against rice blast disease, produced by root-colonizing *B*. *velezensis* to the benefit of its host [31]. Further work is required to corroborate the outcomes of LC-MS analysis, using the authentic compounds as controls to observe all peaks with their fragmentations under the same conditions.

The second line of investigation was launched to assess the xenobiotic metabolizing potential of isolates in the bacterial collection. As the above-mentioned example of *B*. *velezensis* demonstrates, bacterial SMs may be useful not only as medicinal products targeting human or veterinary diseases, but also as bioactive agents protecting crops from devastating pathogens [52, 53]. Chemical warfare in the plant microbiome may involve not just the competing bugs, but also the plant host itself. In this battle, a SM released by one fighter may be subject to detoxification by another. An elegant example was described by Bacon and colleagues [54] for maize and its two endophytic microbes *Fusarium verticillioides* (a pathogenic fungus) and *Bacillus mojavensis* (a beneficial bacterium). Maize produces BOA (a γ-lactam) to kill the fungus, which in turn survives by recruiting a lactamase to convert BOA to 2AP (an aniline) that is eventually inactivated via *N-*malonylation by a specialized NAT enzyme homolog [55–57]. In this race, the bacterium sides with the plant, enhancing the accumulation and subsequent oxidation of 2AP to its orange-colored derivative 2-amino-3H-phenoxazin-3-one (APO) which is more toxic to the fungus than BOA [54]. Apart from their roles as natural pesticides, BOA, 2AP and APO are also used in the synthesis of pharmaceutical and other industrial products [34, 35, 58]. Also of industrial utility is 2-acetamidophenol, i.e. the chemically or microbially synthesized *N-*acetylated form of 2AP [59]. In view of our interest in the BOA-2AP detoxification pathway [57], we screened the bacterial collection against those two compounds, aiming to identify isolates that might prove useful either in the agricultural context as biocontrol agents similar to *B*. *mojavensis*, or as effective detoxifiers of the corresponding synthetic substances. Several *Firmicutes*, *Gammaproteobacteria* and *Streptomycetes* appeared tolerant of a high (1000 μg/mL) concentration of BOA. Although more sensitive, *Alphaproteobacteria* and *Betaproteobacteria* converted BOA to colored products (presumably 2AP and APO), and this was also evident for some *Gammaproteobacteria* (most notably #118- *Enterobacter hormaechei subsp*. *hoffmannii* 170518_3 and #121-*S*. *marcescens* 170518_6), suggesting the enzymatic action of lactamases. As expected, 2AP proved considerably more toxic than BOA, tolerated mainly by *Gammaproteobacteria* with intense pigmentation observed for cultures of *E*. *hormaechei* and *S*. *marcescens*.

While *T*. *paurometabola* DSM 20162^T^ appeared sensitive to BOA and 2AP, it has demonstrated robustness towards 3,4-DCA, a man-made pesticide residue abundant in farmlands [37]. Elimination of aromatic amines, particularly chloroanilines like 3,4-DCA, has been very challenging, as those compounds can be toxic and resistant to microbial biodegradation [60, 61]. Understanding the microbial metabolic pathways associated with arylamine detoxification and/or degradation has attracted interest [62], and NAT-mediated conjugation has been investigated as a route to reducing toxicity of 3,4-DCA in soil [63, 64]. We became interested in *T*. *paurometabola*, as it was found to possess an unusual NAT homolog which, in its recombinant form *in vitro*, demonstrated selectivity towards multiple acyl-CoA donor substrates, particularly malonyl-CoA [37]. Here, challenge of the bacterium *in vivo* with 3,4-DCA generated only the *N-*acetylated conjugate of the compound in the culture medium, detectable by LC-MS. This is consistent with the variable donor substrate selectivity observed for the recombinant NAT enzyme of *T*. *paurometabola* DSM 20162^T^, which may depend on the chemical nature of the acceptor aniline. A similar screen is underway for other bacteria of the collection, in order to identify isolates capable of detoxifying 3,4-DCA and other toxic arylamines.

## Conclusion

This project has aimed to introduce a bacterial collection comprising taxonomically representative isolates that will be useful for "pipeline" laboratory investigations of microbial secondary and xenobiotic metabolism, or other biological functions of interest. We have demonstrated utility of this collection by launching two parallel lines of investigation and providing "proof-of-concept" examples. Co-culture of microbes is recognized as an effective way to activate cryptic biosynthetic pathways, with research guided by the *in silico* prediction of BGCs and their respective chemical products. On the other hand, bioremediation is viewed as an effective, relatively inexpensive and environmentally friendly approach to combat pollution, accelerated through the systematic research of microbial xenobiotic metabolism. Study of the collection in the long term will allow further insights into such processes, identifying strains and metabolites that can be exploited in various biotechnological applications.

## Supporting information

S1 File Bacterial isolates in the study and details of experimental processes and results. (XLS)

S2 File MIBiG results. (XLS)
